# Simulation-based suggestions to improve ibuprofen dosing for patent ductus arteriosus in preterm newborns

**DOI:** 10.1007/s00228-018-2529-y

**Published:** 2018-07-28

**Authors:** Robert B. Flint, Rob ter Heine, Edwin Spaans, David M. Burger, Johan C. A. de Klerk, Karel Allegaert, Catherijne A. J. Knibbe, Sinno H. P. Simons

**Affiliations:** 1 Department of Pediatrics, Division of Neonatology, Erasmus University Medical Center – Sophia Children’s Hospital, Wytemaweg 80, 3015 CN Rotterdam, The Netherlands; 2 Department of Pharmacy and Radboud Institute of Health Sciences (RIHS), Radboudumc, Nijmegen, The Netherlands; 3 Department of Pharmacy, Erasmus University Medical Center, Rotterdam, The Netherlands; 4 Department of Pediatric Surgery, Erasmus University Medical Center – Sophia Children’s Hospital, Rotterdam, The Netherlands; 5 Department of Development and Regeneration, KU Leuven, Leuven, Belgium; 6 Leiden Amsterdam Center for Drug Research (LACDR), Division of Pharmacology, LACDR, Leiden University, Leiden, The Netherlands; 7 Department of Clinical Pharmacy, St Antonius Hospital, Nieuwegein, The Netherlands

**Keywords:** Ibuprofen, Preterm newborn, Patent ductus arteriosus, New dosing regimen, Simulations

## Abstract

**Purpose:**

Ibuprofen is the drug of choice for treatment of patent ductus arteriosus (PDA). There is accumulating evidence that current ibuprofen-dosing regimens for PDA treatment are inadequate. We aimed to propose an improved dosing regimen, based on all current knowledge.

**Methods:**

We performed a literature search on the clinical pharmacology and effectiveness of ibuprofen. (R)- and (S)-ibuprofen plasma concentration-time profiles of different dosing regimens were simulated using a population pharmacokinetic model and evaluated to obtain a safe, yet likely more efficacious ibuprofen exposure.

**Results:**

The most effective intravenous ibuprofen dosing in previous clinical trials included a first dose of 20 mg kg^−1^ followed by 10 mg kg^−1^ every 24 h. Simulations of this dosing regimen show an (S)-ibuprofen trough concentration of 43 mg L^−1^ is reached at 48 h, which we assumed the target through concentration. We show that this target can be reached with a first dose of 18 mg kg^−1^, followed by 4 mg kg^−1^ every 12 h. After 96 h postnatal age, the dose should be increased to 5 mg kg^−1^ every 12 h due to maturation of clearance. This twice-daily dosing has the advantage over once-daily dosing that an effective trough level may be maintained, while peak concentrations are substantially (22%) lower.

**Conclusions:**

We propose to improve intermittent ibuprofen-dosing regimens by starting with a high first dose followed by a twice-daily maintenance dosing regimen that requires increase over time and should be continued until sufficient effect has been achieved.

**Electronic supplementary material:**

The online version of this article (10.1007/s00228-018-2529-y) contains supplementary material, which is available to authorized users.

## Introduction

Patent ductus arteriosus (PDA) is a potentially very harmful condition in the youngest preterm infants, especially in those born before 28 weeks of gestation [1]. PDA has been associated with a range of adverse outcomes including chronic lung disease, necrotizing enterocolitis, intraventricular hemorrhage, and death [2–4].

Twenty years ago, Varvarigou et al. first reported about the effectiveness of COX-2 inhibition with early ibuprofen treatment for human preterm infants with PDA [5]. Subsequently, ibuprofen has been dosed and licensed as once daily on three consecutive days at 10 mg kg^−1^, 5 mg kg^−1^, and 5 mg kg^−1^ day^−1^ via intravenous infusion. This dosing regimen leads to closure of the ductus arteriosus in only about 60% of patients [6–9]. Despite the large number of studies on ibuprofen for PDA, an optimal and widely accepted dosing regimen is still lacking. Generally, increased effectiveness has been reported using higher dosages, although the results of reports on the same dosing regimens and comparable cohorts are very divergent [10–18]. Until now, only Desfrere et al. [10] performed an ibuprofen dose-finding study starting in early neonatal life in which they found a clear difference with lower effectiveness at lower gestational age (GA), although this has not yet led to an adapted dosing regimen. Furthermore, three randomized controlled trials have shown more effectiveness for ibuprofen treatment compared with placebo [18], and for high versus low ibuprofen dosage [10, 15]. Recently, a state-of-the-art meta-analyses of randomized controlled trials comparing pharmacotherapeutic interventions by Mitra et al. in the JAMA came to the same conclusions, and besides found higher effectiveness for oral compared to intravenous administration [19]. In current practice, local interpretation of available evidence has led to a large variety of dosing regimens in clinical practice [20]. To avoid further unnecessary blood sampling in this vulnerable population, we aimed to study available data from literature, PK models, and simulation to suggest improved ibuprofen doses.

In the absence of sufficient evidence, the approach for an optimized ibuprofen therapy should take into account the physiological mechanism that causes active ductal constriction. After term delivery, reduced prostaglandin E2 (PGE2) levels are sensed by the PGE2 receptors (EP4) and promote further constriction of the ductus. Consequently, it is assumed that closure of a patent ductus arteriosus can be enhanced pharmacologically. Inhibition of cyclooxygenase-2 (COX-2) reduces PGE2 generation from arachidonic acid. Intravenous ibuprofen is commercially available as a racemic mixture of (R)- and (S)-ibuprofen. The (S)-enantiomer acts through competition for COX-2, followed by a reversible binding and inhibition. (R)-ibuprofen is a relatively weak inhibitor of COX-2 [21]. The metabolism of ibuprofen has been shown to mature during early life [12, 22], with CYP2C8 mainly responsible for the metabolism of (S)-ibuprofen, and CYP2C9 for (R)-ibuprofen. The mean half-lives for (S)- and (R)-ibuprofen in preterm infants of about 34 h and 8 h at birth, respectively [23], followed by a very rapid increase of (R)-ibuprofen elimination during the first days of life. Furthermore, a spontaneous unidirectional inversion has been described of 63% of (R)- into (S)-ibuprofen in the human body [24]. For durable effect, the (S)-ibuprofen concentration at the COX-2 receptor should remain above a minimal effective concentration [25]. This is confirmed by an increased effectiveness of continuous versus intermittent treatment [13], which has also been reported for oral versus intravenous administration [9, 11, 26, 27]. It seems therefore of relevance to identify a target trough concentration for ductal closure, taking gestational and postnatal age into account. Further, peak concentrations should be minimized regarding safety and toxicity [28, 29]. Namely, the risk of developing side effects and toxicity of nonsteroidal anti-inflammatory drugs seems related to peak concentrations, as continuous administration of indomethacin showed less side effects than an intermittent regimen [29]. In addition, the meta-analyses by Mitra et al. 2018 found that a continuous infusion of intravenous ibuprofen was associated with the lowest incidence of oliguria compared to all included intermittent dosing regimens [19].

Furthermore, the high remaining proportion of patent open ducts after 3 days of ibuprofen treatment proves that inhibitory (S)-ibuprofen concentration was too low or treatment was too short. Yet, if ibuprofen treatment is continued, large differences exist between neonatal intensive care units (NICUs) on whether to use the same dosage or to increase ibuprofen dosing with age, so to adjust for the increasing ibuprofen clearance due to maturation.

In this study, we combine evidence on the effect of various intravenous ibuprofen-dosing regimens on PDA closure, with a previously developed population PK model. Additionally, we suggest an improved ibuprofen-dosing regimen.

## Methods

### Effectiveness of ibuprofen

Considering ibuprofen’s mechanism of action with competitive, reversible COX-2 binding [25], we aim to maintain a minimal (S)-ibuprofen concentration for optimal effectiveness. In this simulation study, a target trough concentration (S)-ibuprofen was determined from simulations of (R)- and (S)-ibuprofen plasma concentration-time profiles following the most effective reported intravenous dosing regimen. The latter has been recently reported in a state-of-the-art meta-analyses by Mitra et al. in the JAMA incorporating all reported pharmacotherapeutic interventions for PDA closure.

### Pharmacokinetic model

Published population pharmacokinetic (PK) models of ibuprofen in preterm infants were investigated using PubMed with MeSH terms: “Ibuprofen,” “pharmacokinetics,” and “infant newborn”. The population PK models (see Supplementary File 1) were compared with respect to birth weight, gestational age (GA), and postnatal age (PNA) of the cohort, ibuprofen-dosing regimen, route of administration, and studied ibuprofen enantiomers [12, 22, 23, 30, 31].

### Simulations

In order to illustrate our suggestions for dosage improvements, we simulated plasma concentration-time profiles of (R)- and (S)-ibuprofen after several intravenous dosing regimens for a typical neonate with PDA and a body weight of 840 g, which we determined from the cohort of all preterm newborns with a significant PDA of the Erasmus Medical Center [32]. First ibuprofen dosage was administered at PNA of 24 h as an early start has shown to be more effective than a late start.

Simulations were performed using Nonlinear Mixed Effects Modeling (NONMEM, version 7.3, Globomax LLC, Ellicott City, Maryland, USA) based on Gregoire et al. [23]. For simulation of intermittent dosing regimens, ibuprofen was administered intravenously in 15 min. Higher infusion rates would not be recommended to avoid high peak levels and fluid overload.

We compared the population predicted trough plasma concentrations of (S)-ibuprofen at 48 h after start of different dosing regimen, which concerned frequently reported regimens as well as new dosing proposals. Furthermore, we compared the peak concentrations as these may be related to safety and toxicity [28, 29]. For the purpose of predicting a peak concentration, a time point at 48.5 h after start of ibuprofen therapy was chosen, as this was the latest dose that was administered in 15 min at 48 h in all regimens, incorporating 15 min after infusion to allow drug distribution.

## Results

### Effectiveness of ibuprofen

**Fig. 1:**
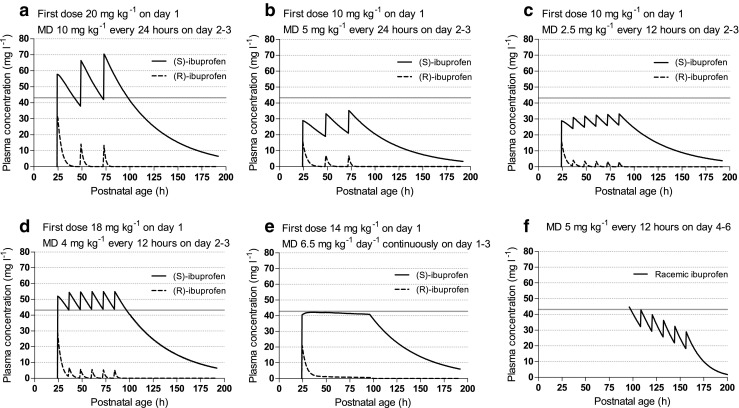
Simulations on population-predicted plasma concentration-time profile of (R)- and (S)-ibuprofen. Population predictions without inter-patient variability were performed for one typical neonate with a body weight of 840 g. A gray reference line indicates the (S)-ibuprofen target concentration. In Figs. a–e: Plasma concentrations of the separate (R)- and (S)-enantiomers were simulated with the PK model of Gregoire et al. [23] starting at a postnatal age of 24 h. In Fig. f: Plasma concentrations of racemic ibuprofen ((R) and (S) not separately) were simulated with the PK model of Hirt et al. [12] starting at a postnatal age of 96 h. Abbreviation: *MD* maintenance dose

The most effective intravenous ibuprofen-dosing regimen determined in meta-analyses by Mitra et al. consisted of a 20 mg kg^−1^ first dose, followed by additional 10 mg kg^−1^ doses after 24 and 48 h, all with 15 min infusion rates [19]. Simulation of concentration-time profiles of both ibuprofen enantiomers in a typical neonate with PDA at PNA of 24 h and a body weight of 840 g following this dosage regimen, predicted a corresponding (S)-ibuprofen trough concentration at 48 h after ibuprofen start of 43 mg l^−1^ (Fig. 1a). Thus, for this neonate, we hypothesized in our study that an (S)-ibuprofen target concentration of 43 mg l^−1^ will achieve optimal effectiveness for a preterm infant with a body weight of 840 g starting ibuprofen at 24 h PNA.

### Pharmacokinetic model

From the five reported population PK models on ibuprofen in preterm born infants (see Supplementary File 2), the model by Gregoire et al. [23] was selected, as the model was based on the largest, and best matching cohort: 108 premature infants, with a median birth weight of 880 g (range 300–1700), median GA of 26.9 weeks (range 24.0–30.7), median PNA of 1 day at ibuprofen start (range 0–8), and described the PK of (R)- and (S)-ibuprofen adequately for intravenous ibuprofen administration of 5 to 10 mg kg^−1^ day^−1^. Their model consisted of a one-compartment model for both (R)- and (S)-ibuprofen, with unidirectional bioconversion of (R)-ibuprofen into (S)-ibuprofen, and the effect of increasing elimination of the (R)-enantiomer with increasing PNA, being the single covariate in the model. The pharmacokinetic parameter estimates are shown in Supplementary File 3.

### Simulations

Population predictions of plasma concentration-time profiles of (R)- and (S)-ibuprofen for a neonate at PNA 24 h and body weight of 840 g are shown in Fig. 1 for several dosages. Table 1 gives an overview of the simulation results following different dosing regimen. This illustration allowed to compare the dosing regimen with respect to the total dose of ibuprofen administered, C_min_, C_max_. Comparison of Fig. 1b with Fig. 1c visualizes that dividing an equal daily dose of 5 mg kg^−1^ day^−1^ from one to two administrations, increased the trough concentration from 21 to 25 mg l^−1^ at 48 h after start, and lowered the peak concentration from 34 to 31 mg l^−1^. The determined target concentration of 43 mg l^−1^ for (S)-ibuprofen was reached with a first dose of 18 mg kg^−1^, followed by 4 mg kg^−1^ every 12 h (8 mg kg^−1^ day^−1^) (Fig. 1d). Table 1 and Fig. 1 allow a comparison of our most optimal simulated dosing with the most effective reported regimen of 20-10-10 as is known from previous clinical trials. We show that comparable trough concentrations were reached with a 5% lower cumulative 3-day dosing, and 22% lower peak concentrations. To reach the same target (S)-ibuprofen concentration with continuous infusion, a first dose of 14 mg kg^−1^ followed by a maintenance dose of 6.5 mg kg^−1^ day^−1^ was required (Fig. 1e). This regimen led to the lowest total 3-day dose, and the lowest peak concentration. Simulations upon dosing ibuprofen in neonates with a PNA of 96 h using the racemic-ibuprofen PK model of Hirt et al. showed that an increased maintenance dosage was required to 5 mg kg^−1^ every 12 h (10 mg kg^−1^ day^−1^) (Fig. 1f). These simulations illustrate our proposal to start with a first dose followed by a maintenance dose in twice daily that requires increased dosing over time, and should be continued until sufficient effect has been achieved or treatment needs to be terminated for other reasons.

**Table 1:** Population predicted (S)-ibuprofen concentrations following simulation of various intravenous ibuprofen-dosing regimens

	First dose (mg kg^−1^)	MD (mg kg^−1^)	Duration (days)	Cumulative dose (mg kg^−1^)	Pop pred C_trough_ T48 after start (mg l^−1^)	Pop pred C_peak_ T48.5 after start (mg l^−1^)
a	20	10 every 24 h	3	40	42.5	70.4
b	10	5 every 24 h	3	20	22.2	35.2
c	10	2.5 every 12 h	3	22.5^#^	26.5	32.9
d	18	4 every 12 h	3	38 ^#^	43.8	54.9
e	14	6.5 continuously	3	27	40.9	41.4

Simulations for a typical neonate with PDA with body weight 840 g, and PNA of 24 h

^#^On day 1, the first dose was followed by the first maintenance dose at 12 h

*MD* maintenance dose, *Pop pred* population predictions, *T* time after start of ibuprofen therapy

## Discussion

Based on reported effectiveness of ibuprofen and its mechanism of action, we suggest a first loading dose followed by a relatively high intravenous dosage, in twice daily, which is further increased with postnatal age, and continued until ductal closure has been achieved. In our simulation study that needs further validation first, we illustrate a dosage proposal for an intravenous ibuprofen-dosing regimen for a typical neonate with PDA with birth weight 840 g at PNA 24 h, to start with a first dose of 18 mg kg^−1^, followed by 4 mg kg^−1^ every 12 h. Above 96 h, the dose should be increased to 5 mg kg^−1^ every 12 h until sufficient effect has been achieved or treatment needs to be terminated due to side effects, contraindications, or insufficient effect. Thus, COX-2 may be sufficiently inhibited, without exposing preterm infants to unnecessarily high concentrations.

We combined all available evidence on the effectiveness of intravenous ibuprofen-dosing regimens on PDA closure and on the mechanism of action, with a previously developed (R)/(S)-ibuprofen population pharmacokinetic model [23]. Herewith, we are the first in proposing to maintain a certain (S)-ibuprofen target concentration. Although, the height of the target is most certainly different with gestational and postnatal age, our approach is supported by the reported in vitro inhibitory (S)-ibuprofen concentration for COX-2 inhibition leading to 90% reduction of the agonistic effect of PGE2 (IC_90_) [30, 33, 34]. Only the unbound (S)-ibuprofen concentration is able to have an effect on the ductus arteriosus. Aranda et al. found > 99% protein binding of (S)-ibuprofen in adult blood compared to 94% in neonates [30]. Neupert et al. found an IC_90_ for unbound (S)-ibuprofen of 2.1 mg l^−1^ [34]. Assuming an unbound fraction of 6%, a total (S)-ibuprofen concentration of around 35 mg l^−1^ will achieve the IC_90_ for COX-2 inhibition. This finding is in line with our proposed target plasma concentration for (S)-ibuprofen of 43 mg l^−1^.

We propose to divide the daily dose from one to two administrations, either for safety or to increase effectiveness. The risk of developing side effects and toxicity of nonsteroidal anti-inflammatory drugs seems related to peak concentrations. The meta-analyses by Mitra et al. reported less side effects with a continuous administration than an intermittent regimen, although the odds ratio for oliguria of 0.07 was not found significant (0.00–1.84) [19]. Gournay et al. reported a placebo controlled trial with major side effects of ibuprofen concerning gastrointestinal adverse effects, severe intraventricular hemorrhages caused by thrombocytopathy, necrotizing enterocolitis, and renal dysfunction [18]. The former has also been illustrated by De Cock et al. [35] reporting a 16% reduced clearance of the renally eliminated amikacin due to combination with ibuprofen. Our proposed maintenance dosage of 4 mg kg^−1^ every 12 h, leads to comparable trough concentrations with 10 mg kg^−1^ day^−1^ every 24 h, but with 22% lower peak concentrations. Secondly, continuous ibuprofen administration has been shown to be more effective than intermittent by Lago et al. [13] even without a loading dose, leading to 84 vs 64% PDA closure, respectively. The higher responsiveness following a more stable ibuprofen exposure is confirmed by the counterintuitive finding of higher closure rates following oral than intravenous administration, which is thought to be caused by the more graduate absorption following oral intake [36]. The meta-analyses by Mitra et al. also concluded that oral administration is the most effective treatment, followed by high-dose intermittent IV dosing regimen [19]. Nevertheless, oral administration is often not tolerated during the first postnatal days of an extremely preterm born infant. Continuous ibuprofen showed low effectiveness in the meta-analyses, probably due to the absence of a loading dose and relatively low maintenance. Although an adequately dosed continuous infusion preceded by a loading dose would be highly effective, an intermittent regimen is preferred. A continuous infusion has multiple limitations, e.g., requiring a continuously available intravenous catheter, physiochemical incompatibility with intravenous co-medication, and increased risk for infections. Altogether, for an intermittent regimen we propose to divide the daily dose in two administrations which allows to safely increase the daily dose and trough plasma concentrations of (S)-ibuprofen with limited increase of peak plasma concentrations.

Since Varvarigou et al. first published on ibuprofen for PDA, most reported trials considered ibuprofen a 3-day course, which may be repeated once or twice in clinical practice and seems to improve outcome after initial failure [37–39]. Awaiting the result of echocardiography on day 4, generally, no ibuprofen is administered and often will not be restarted until the next day in case of insufficient ductal closure. This delay in ibuprofen administration leads to an undesirable drop of (S)-ibuprofen plasma concentration on the fourth day of treatment, and an unnecessary high peak concentration following the new loading dose with the start of an additional 3-day course. The success of an additional course has been shown but we considered this regimen with additional 3-day courses suboptimal and potentially unsafe. Instead, we suggest to continue ibuprofen uninterrupted, and therefore maintain the COX-2 inhibition until sufficient closure of the ductus arteriosus is achieved, or until ibuprofen treatment needs to be terminated due to side effects, contraindications, or insufficient effect. However, we were not able to propose a reliable dosage above 96 h PNA using the PK model by Gregoire, due to the absence of a covariate reflecting maturation of clearance of (S)-ibuprofen. Namely, considering the rapid maturation of (S)-ibuprofen’s CYP2C9 metabolism [40], and an increased clearance is highly expected and has been described by Hirt et al. in a cohort with median PNA of 69 h [12]. In addition to the suggested improvements following a higher dose, considering the large interindividual variability in neonates and mechanism of action, one may also argue that further dose tailoring, e.g., with TDM, may be of added value. Although this off course would first require a validated target concentration in clinical practice. Taking these findings into account, we propose to increase the daily dosage above a PNA of 96 h, to 5 mg kg^−1^ every 12 h, as this is the highest, and still safe, investigated daily dose in preterm infants (Fig. 1f).

Our approach provides important lessons and allows a unique dose comparison, but is limited by some assumptions we made regarding effectiveness, the target concentration, the performance of the population PK model [23], as well as the sparse knowledge on safety. The large variability in published success rates may partly be caused by maturation; i.e., increased spontaneous closure of the ductus with GA [41], or increased ibuprofen clearance with GA and PNA leading to more subtherapeutic concentrations [10, 12]. In confirmation, Desfrere et al. reported 77% ductal closure following a first dose of 10 mg kg^−1^ followed by 5 mg kg^−1^ on days 2 and 3 in patients with GA of 27–29 weeks, compared to success in 31% of neonates below 27 weeks of gestation [10]. Further, a target for ibuprofen effectiveness on PDA closure has not yet been determined in clinical trials. If such a target would be available, we would suggest therapeutic drug monitoring in the individual patient. Our simulated dosages would be a good starting point, with further dose adaptations in the individual patient based on bed-side determined plasma levels in the near future. Although we considered the PK model by Gregoire et al. as the best model available for simulations at PNA of 24 h, the model does not scale (S)-ibuprofen PK on bodyweight nor does it incorporate GA and PNA as a covariate for (S)-ibuprofen. Neither does the model allow simulations following oral administration of ibuprofen, which has been reported with remarkably higher effectiveness compared to intravenous dosing regimens; 83% versus 62%, respectively [14, 27]. Although, oral administration to an extremely preterm born infant is not possible on day 1 after birth due to feeding intolerance, it seems an attractive alternative route of administration. Furthermore, we assumed that the ibuprofen PK is linear over a 5–20 mg kg^−1^ dose range, while linearity has only been explored within the 5–10 mg kg^−1^ range for model development. As such, we could not simulate beyond 96 h PNA with the PK model of Gregiore. Finally, safety has not yet been related to ibuprofen dosage and exposure.

Concluding, currently used ibuprofen-dosing regimens for PDA seem suboptimal. Based on the best evidence thus far available, we suggest that if decided to treat the PDA intravenously, a high dosage should be used in twice daily, increased with postnatal age, and continued until ductal closure has been achieved. For illustration, a typical neonate with birth weight 840 g at PNA 24 h may start with an ibuprofen first dose of 18 mg kg^−1^, followed by a maintenance dose of 4 mg kg^−1^ every 12 h until 96 h PNA. Above 96 h, a dose increase is suggested to 5 mg kg^−1^ per dose every 12 h until sufficient effect has been achieved or treatment needs to be terminated due to side effects, contraindications, or insufficient effect. Thereby, sufficient inhibition of COX-2 may be achieved and maintained, without exposing preterm infants to unnecessarily high (S)-ibuprofen peak concentrations that have not been proven safe. These suggestions should be incorporated in current ibuprofen dosing regimens and require a prospective evaluation, allowing to bridge the remaining gaps concerning treatment outcome of different administration routes, maturation of spontaneous closure, safety of ibuprofen dosing regimens, and covariates for (S)-ibuprofen PK. Finally, placebo controlled studies are warranted to characterize dynamics of natural PDA closure and to quantify drug-related effectiveness.

## Electronic supplementary material

ESM 1 (DOCX 35 kb)
