# Supplementary material for: Simulation-based suggestions to improve ibuprofen dosing for patent ductus arteriosus in preterm newborns
Source: Eur J Clin Pharmacol. 2018 Jul 28;74(12):1585–91. doi: 10.1007/s00228-018-2529-y (PMC6244763; doi:10.1007/s00228-018-2529-y)
Supplement: Supplementary file 1 — (DOCX 35 kb) [file 228_2018_2529_MOESM1_ESM.docx]

**SUPPLEMENTARY FILES**

**Title manuscript: Simulation-based suggestions to improve ibuprofen dosing for patent ductus arteriosus in preterm newborns**

Journal: European Journal of Clinical Pharmacology

Robert Flint^1,2,3^, Rob ter Heine^2^, Edwin Spaans^1^, David Burger^2^, Johan de Klerk^1^, Karel Allegaert^4,5^, Catherijne Knibbe^6,7^, Sinno Simons^1^

1. Department of Paediatrics, Division of Neonatology, Erasmus University Medical Centre – Sophia Children’s Hospital, Rotterdam, The Netherlands
2. Department of Pharmacy and Radboud Institute of Health Sciences (RIHS), Radboudumc, Nijmegen, The Netherlands
3. Department of Pharmacy, Erasmus MC, Rotterdam, The Netherlands
4. Department of Paediatric Surgery, Erasmus University Medical Centre – Sophia Children’s Hospital, Rotterdam, The Netherland
5. Department of Development and Regeneration KU Leuven, Leuven, Belgium
6. Leiden Amsterdam Center for Drug Research (LACDR), Division of Pharmacology, LACDR, Leiden University, Leiden, The Netherlands
7. Department of Clinical Pharmacy, St Antonius Hospital, Nieuwegein, The Netherlands

**Corresponding author**

Robert Flint

Erasmus Medical Center - Sophia Children’s Hospital

Department of Pediatrics, Division of Neonatology

Wytemaweg 80

3015 CN Rotterdam

The Netherlands

Email: r.flint@erasmusmc.nl

Phone: +31 10 7036582

**Supplementary File 1. Pharmacokinetic models**

The second best PK model for our aim was reported by Hirt et al. [1], who found a significant increase of racemic-ibuprofen clearance with increasing PNA. The major limitations were a high median PNA at start of 69 hours which makes the model unsuitable for simulations in the first days of life, and the lack of discrimination between both enantiomers. Therefore, this PK-model can only be used for a suggested dosing regimen after 96 hours. The three other PK-models were judged less suitable: Overmeire et al. [2] included a small cohort of 27 infants with a relatively high median GA of 28.6 weeks and a high median PNA of 69 hours; Aranda et al. [3] studied an even smaller cohort of 21 preterms; the cohort used for the model of Gregoire et al. 2004 [4], was also part of the model that we selected [5].

**Supplementary File 2. Overview of reported ibuprofen population PK models in neonates.**

|  | **Cohort descriptives**  N, BW (g), GA (weeks),  PNA at start (hours) | **Route of administration,**  **Dosage** | **Analytes,**  **PK parameter estimates, covariates** | **Limitations** |
| --- | --- | --- | --- | --- |
| Aranda et al.  1997 [3] | N=21  BW mean = 945 (range 575-1450)  GA mean = 26.8 (range 22-31)  PNA = range 0-3 | Intravenous  LD: 10 mg kg^-1^  MD: 5 mg kg^-1^ day^-1^  in 1 dose | Total racemic  CL_rac-ibu_: 2.1 ml kg^-1^ h^-1^  Vd_rac-ibu_: 62.1 ml kg^-1^  No covariates | Small cohort  Total rac-ibuprofen  No covariates |
| Overmeire et al.  2001 [2] | N=27  GA mean = 28.6 (SD 1.9)  BWmean = 1250 (SD 460)  PNA median = 72 | Intravenous  LD: 10 mg kg^-1^  MD: 5 mg kg^-1^ day^-1^  in 1 dose | Total racemic  CL_rac-ibu_: 10.8 ml h^-1^  Vd_rac-ibu_: 357 ml kg^-1^  No covariates | High PNA and GA  Total ibuprofen  Small cohort  Standard 2-stage  No covariates |
| Gregoire et al.  2004 [4] | N=62  GA mean = 26.6 (range 24.0-27.9)  BW mean = 855 (range 300-1320)  PNA range = 0-6 | Intravenous  LD: 10 mg kg^-1^  MD: 5 mg kg^-1^ day^-1^  in 1 dose | Separate (R)/(S)-ibuprofen  CL_(S)-ibu_: 5.0 ml h^-1^  CL_(R)-ibu at birth_: 12.7 ml h^-1^  Vd_(S)-ibu & (R)-ibu_: 183 ml kg^-1^  Covariates: GA on CL_(R)- & (S)-ibu_ | Smaller cohort than PK model in 2008 by Gregoire et al. |
| Gregoire et al.  2008 [5] | N=108  GA median = 26.9 (range 24.0-30.7)  BW median = 880 (range 300-1700)  PNA median = 24 (range 0-192) | Intravenous  LD: 10 mg kg^-1^  MD: 5 mg kg^-1^ day^-1^  in 1 dose | Separate (R)/(S)-ibuprofen  CL_(S)-ibu_: 3.5 ml h^-1^ kg^-1^  CL_(R)-ibu at birth_: 25.5 ml h^-1^ kg^-1^  Vd_(S)-ibu_: 173 ml kg^-1^  Vd_(R)-ibu_: 306 ml kg^-1^  Covariates: PNA on CL_(R)-ibu_ | No covariate for PNA on CL_(S)-ibu_  Low PNA |
| Hirt et al.  2008 [1] | N=66  GA median = 28 (range 25-34)  BW median = 1015 (range 490-1986)  PNA median = 69 (range 14-262) | Intravenous  LD: 10 mg kg^-1^  MD: 5 mg kg^-1^ day^-1^  in 1 dose | Total racemic  CL_rac-ibu_: 9.49 ml h^-1^  Vd_rac-ibu_: 360 ml kg^-1^  Covariate: PNA on CL_rac-ibu_ | Total ibuprofen  High PNA at start  High GA |

Abbreviations: GA: gestational age; PNA: postnatal age; BW: birth weight; SD: Standard Deviation; LD: loading dose; MD: maintenance dose; CL: Clearance; Vd: Volume of distribution; Rac: Racemic

**Supplementary File 3. Pharmacokinetic parameter estimates of model Gregoire et al. 2008 [5].**

|  | **Parameter** | **Population mean** | **Interindividual variability, CV% (95% CI)** |
| --- | --- | --- | --- |
| **S-ibuprofen** | Kel1, h^-1^ (95% CI) | 0.020 (0.017 - 0.024) | 58 (38 - 73) |
|  | V1, mL kg^-1^ (95% CI) | 173 (156 - 190) | 26 (19 - 32) |
|  | CL_S_, mL h^-1^ kg^-1^ | 3.5 |  |
|  | T_1/2 S_, h | 34.3 |  |
| **R-ibuprofen** | Kel2, h^-1^ (95% CI) | 0.069 (0.046 - 0.093) | 26 (0 - 38) |
|  | Ɵ_PNA_, h^-1^ per postnatal day ^a^ (95% CI) | 0.155 (0.133 - 0.177) |  |
|  | K21, h^-1^ (95% CI) | 0.014 (-0.006 - 0.034) |  |
|  | V2, mL kg^-1^ | 306 (240 - 372) | 95 (58 - 121) |
|  | CL_R at birth_, mL h^-1^ kg^-1^ | 25.5 |  |
|  | T_1/2 R_ _at birth_, h | 8.3 |  |

Kel1 and Kel2 indicated elimination micro-constants of (R)- and (S)-ibuprofen; V1 and V2, volumes of distribution of (R)- and (S)-ibuprofen; K21, bioconversion micro-constant from (R)- to (S)-ibuprofen; CL_R_ and CL_S_, clearance of (R)- and (S)-ibuprofen

a. Kel2 = Kel2 at birth + Ɵ_PNA*_postnatal age.

Abbreviations: CI: Confidence interval

1 Hirt D, Van Overmeire B, Treluyer JM, Langhendries JP, Marguglio A, Eisinger MJ, Schepens P, Urien S (2008) An optimized ibuprofen dosing scheme for preterm neonates with patent ductus arteriosus, based on a population pharmacokinetic and pharmacodynamic study. Br J Clin Pharmacol 65 (5): 629-636 10.1111/j.1365-2125.2008.03118.x

2 Van Overmeire B, Touw D, Schepens PJ, Kearns GL, van den Anker JN (2001) Ibuprofen pharmacokinetics in preterm infants with patent ductus arteriosus. Clin Pharmacol Ther 70 (4): 336-343 S0009-9236(01)13478-8 [pii]

3 Aranda JV, Varvarigou A, Beharry K, Bansal R, Bardin C, Modanlou H, Papageorgiou A, Chemtob S (1997) Pharmacokinetics and protein binding of intravenous ibuprofen in the premature newborn infant. Acta Paediatr 86 (3): 289-293

4 Gregoire N, Gualano V, Geneteau A, Millerioux L, Brault M, Mignot A, Roze JC (2004) Population pharmacokinetics of ibuprofen enantiomers in very premature neonates. Journal of clinical pharmacology 44 (10): 1114-1124 10.1177/0091270004268320

5 Gregoire N, Desfrere L, Roze JC, Kibleur Y, Koehne P (2008) Population pharmacokinetic analysis of Ibuprofen enantiomers in preterm newborn infants. Journal of clinical pharmacology 48 (12): 1460-1468 10.1177/0091270008323752
